# Health and welfare in organic livestock production systems—a systematic mapping of current knowledge

**DOI:** 10.1007/s13165-020-00334-y

**Published:** 2020-11-25

**Authors:** Magdalena Presto Åkerfeldt, Stefan Gunnarsson, Gun Bernes, Isabel Blanco-Penedo

**Affiliations:** 1 Department of Animal Nutrition and Management, SLU, Swedish University of Agricultural Sciences, Uppsala, Sweden; 2 Department of Animal Environment and Health, SLU, Swedish University of Agricultural Sciences, Skara, Sweden; 3 Department of Agricultural Research for Northern Sweden, SLU, Swedish University of Agricultural Sciences, Umeå, Sweden; 4 Department of Clinical Sciences, Unit of Veterinary Epidemiology, SLU, Swedish University of Agricultural Sciences, Uppsala, Sweden

**Keywords:** Animal welfare, Organic farming standards, Systematic review, Health indicators

## Abstract

This review aimed to systematically map and summarize the status of animal health and welfare in organic production. The prevalence of diseases and behavioural effects in organic dairy cow, beef cattle, sheep, pig, laying hen and broiler chicken were discussed in the context of the organic values and current knowledge on animal health and welfare. In total 166 peer-reviewed scientific publications between 2008 and 2020 were included. No strong evidence for neither inferior nor distinctly higher animal welfare in organic compared with conventional production could be supported. The welfare status of organic livestock is in general good in relation to the OIE definition of animal health and welfare. However, organic systems are still facing several challenges related to animal health and the arising of goal conflicts due to management and practical implications. Greater possibilities to perform species-specific behaviours in organic production systems, however, indicate that the organic standards offer a good framework for high animal welfare management. For organic dairy farmers, the main health problems are similar to those of non-organic farms; especially mastitis and lameness need improvement. Parasites, together with mastitis and lamb mortality, are important welfare issues in organic sheep production. Piglet mortality, leg problems, parasite load and increasing respiratory problems are of major relevance in organic pig production. For organic laying hens, major health challenges relate to feather pecking and cannibalism, parasites and possibilities to express species-specific behaviours. For organic broilers, dermatitis of footpads, hocks and breast are reported as main health issues.

## Introduction

Organic animal production has experienced a rapid development that also has led to changes in the way the production is conducted. The goals and principles of organic production throughout Europe are well defined, and the marketing of certified organic products is thoroughly regulated by the European Union since the 1990s and thereafter revised accordingly (EU 2018). The production is based on a setup of general principles established by the International Federation of Organic Agriculture Movements (IFOAM 2005). Concerning animal health and welfare, the principle implies the maintenance of physical, mental, social and ecological well-being as well as the absence of diseases. The IFOAM principles are reflected in the European regulation of organic production (EC 2008), which also includes high animal welfare standards, in particular when it comes to meeting animals’ species-specific behavioural needs and protecting their health. The regulation is the fundament for the national certification within the EU member states, even if the flora of certifications may be diverse and differ between countries (Sanders 2013). Although the fundamental rules of organic production are legally defined, organic livestock production covers a broad diversity of production systems varying both between and within countries and animal species. The development of the organic sector, from the early 1970s until today, has resulted in changes in both how the production system is performed as well as the view of the production. From being a movement based on ideological thoughts, it has progressed to be defined as a production method by minimum standards, which limit the possibilities to provide a clear frame to characterize in dissociation to conventional production. Consumers expect high animal health and welfare in organic farming, but there are also doubts whether these systems achieve this better than conventional animal husbandry systems do (Sundrum et al. 2010; Sutherland et al. 2013).

Health and welfare may be defined differently, and these definitions may then have different implications (Gunnarsson 2006). Internationally, the World Organisation for Animal Health (OIE 2019) provides the most accepted definition of animal health and welfare meaning “the physical and mental state of an animal in relation to the conditions in which it lives and dies.” It covers “the five freedoms”: (1) freedom from hunger, malnutrition and thirst; (2) freedom from fear and anxiety; (3) freedom from heat stress or physical discomfort; (4) freedom from pain, injury and disease; and (5) freedom to express normal patterns of behaviour. There is no reason to define animal health and welfare in organic animal husbandry in a different manner than in conventional. However, according to the organic values and understanding of animal welfare, the IFOAM principles focus more and more on the animals’ quality of life and emphasize the emotional state of the animal, as well as the concept of “naturalness” as part of animal welfare. Organic animals should, for example, be provided with conditions for living a natural life in accordance with their physiological and behavioural conditions and well-being, in an environment that most closely resembles that to which the species is evolutionarily adapted. Animals’ ability to live a natural life is thereby considered as a prerequisite for good animal welfare (IFOAM 2005; EU 2018). That is, animal health promotion strategies aim to go beyond targeting specific disease conditions and aim at reaching a state of homeostasis (Vaarst and Alrøe 2012). Within the fundamental ideas of organic production that are based on an ecocentric view, natural living is seen as a value in itself and fulfils a higher rank than the absence of pain and suffering (Lund and Algers 2003). Ecological resilience is considered as a superior goal, and emphasis is put on system-thinking rather than on situations for individual animals (Verhoog 2000), and to reach a natural living, some negative experiences for the individual may be accepted (Verhoog et al. 2004). Sustainable agriculture is seen as multifunctional, where animals provide not only feed but also serve to cultivate farmland to preserve biodiversity and ecosystems services as open landscape. The system as such might thereby imply conflicts with health and welfare (Öhlund et al. 2017), especially between that of the system and that of the individual animal, e.g. leg health or parasite burden of free-ranging animals. This might be an underlying factor to the criticism about animal welfare in organic production, but the criticism may partly also depend on different ethical positions and views and a lack of dialogue between stakeholders (Lund and Algers 2003; Duval et al. 2016; Krieger et al. 2020).

The development of the organic sector and demands of improved animal health and welfare has led to investigations in the field to increase the knowledge and identify risk factors. However, the results are diverse, and some inconsistent interpretations of the outcome of the research might probably depend on differing research objectives and criteria (i.e. comparing organic vs. conventional production systems), heterogeneity of regions, production and farm conditions, inadequate experimental design, as well as the too low number of farms included. The variation within organic production between farms and regions as well as national differences in the interpretation of the regulation also need to be considered when comparing organic livestock systems with each other and with conventional ones (Zoiopoulos and Hadjigeorgiou 2013). Despite an increasing number of epidemiological studies in recent years, where more farm-specific factors have been examined, outcome-oriented animal health and welfare indicators are still requested (Darnhofer et al. 2010; EFSA 2012; Sundrum 2014). Some systematic reviews with comparative assessments of animal welfare with regard to the differences between organic and conventional farming have also been performed (Sundrum 2001; Hovi et al. 2003; Lund and Algers 2003; van Wagenberg et al. 2017). However, the fact that organic livestock systems and management change over time implies continuous revision. It is essential to include a broader discussion about what the reference system should be, with respect to current knowledge on animal health and welfare, legislative rules, consumer and producer expectations, previous and future situations, all in the context of the fundamental organic values, goals and principles.

The general objective of this review was therefore to map and summarize the status of animal health and welfare in organic dairy cow, beef cattle, sheep, pig, laying hen and broiler chicken production systems. The review focuses on prevalence of diseases and behavioural effects for the different animal categories, respectively, with the ambition to discuss the results in the context of the fundamental organic values and current knowledge on animal health and welfare.

## Material and methods

### Definitions of the literature review

The literature review focused on specific health and welfare indicators for different animal categories: dairy cow, beef cattle, sheep, pig, laying hen and broiler chicken. Livestock production not certified according to any organic certification scheme was referred to as conventional (Mie et al. 2017). Year 2008 was set as starting point for the search period as a new commission regulation with detailed rules for the implementation of the organic regulation was applied at that time (EC 2008). The different categories of health and welfare indicators that were chosen to be included in the review were mastitis, metabolic disorders, nutrition deficiencies and digestive disorders, reproductive disorders, foot/hoof/leg disorders, external and internal parasites, respiratory diseases, skin and tail lesions, abscesses, feather pecking and cannibalism and behavioural parameters.

### Search of literature

The review included peer-reviewed scientific literature published between the 1st of January 2008 and the 16th of January 2020. The same search items on four search engines were used: (1) Web of Science Core Collection, (2) CABI, (3) Medline and (4) Scopus. The search was constructed according to a PIO approach (Population, Intervention and Outcome). Specific (for each animal category respectively) and general (common for all animal categories) paragraph search terms were articulated due to the possible search terms typically used in each area. The search terms were defined in collaboration with a professional librarian specialized in scientific databases. Since the word “organic” is used in many different contexts and there is no term consistently and collectively used in the literature for the measures that we wanted to investigate, the effect of the searches had to be quite extensive to capture the relevant papers. The final search terms are presented in Table 1. The search was performed by the same person in one database at a time for the categories “dairy cows and beef cattle”, “sheep”, “pigs”, “laying hens and broiler chickens”, respectively, on two occasions. A first search covered literature between the 1st of January 2008 and the 31st of March 2019. An update of the search was performed on the 16th of January 2020, just before completion of the manuscript, using the same search terms but restricting the search to the time period after the original searches were performed, thus including literature between the 1st of April 2019 and the 16th of January 2020. Relevant references found through the update were included in the review.

**Table 1:** Approach and structured steps used to conduct the systematic search of the literature

Paragraph search terms	Animal category			
	Dairy cows and beef cattle	Sheep	Pigs	Laying hens and broiler chickens
**P**=Population	TS=(bovine or cattle or cow or cows or heifer* or beef* or suckler* or dairy or calve* or calf*)	TS=(Ovine* OR sheep* OR ewe* OR hogget* OR lamb or lambs)	TS=(porcine OR pork OR pig OR pigs OR swine OR "sow" OR "sows" OR gilt* OR piglet*)	TS=(hen or hens or pullet or pullets or chicken or chickens or broiler or broilers or poultry)
**I**=Intervention	TS=((organic or extensive*) NEAR/2 (agricult* or farm* or breed* or "bred" or pasture* or pastor* or system* or rear* or feedstuff or cows or beef or production) or freerange or graz* or roughage or "grass silage" or (housing near/1 system*)	Sheep: TS=((organic OR extensive*) NEAR/2 (agricult* OR farm* OR breed* OR "bred" OR system* OR rear* or production or lamb* or sheep* or mutton or wool or fleece*) OR TS=(pasture* or bedding or bedded or "sheep flock*")	TS=((organic or extensive*) NEAR/2 (agricult* OR farm* OR breed* OR "bred" or pasture* OR pastor* OR system* OR rear* OR feedstuff OR pigs OR pork or production) OR freerange OR graz* OR roughage OR "grass silage" OR (housing near/1 system*))	TS=((organic or extensive*) NEAR/2 (agricult* or farm* or breed* or "bred" or pasture* or pastor* or system* or rear* or feed* or egg or eggs or chicken or poultry or broiler* or hen or production) or freerange or "free range" or molt* or outdoors or veranda or aviar* or perch* or (housing near/1 system*)
**O**=Outcome	TS=( health OR welfare OR disease* OR infection* OR bacteri* OR disorder* OR injurie* OR zoono* OR mortality OR longevity OR liveability OR pathogen* OR phatologic* OR behavio* OR stereotyp* OR culling* OR metabolic* OR perform* OR producti* OR reproducti* OR fertility OR parasite* OR gastrointestin* OR nematode* OR endoparasit* OR ectoparasit* OR trematode* OR "body condition*"			
**O**=Outcome	TS=(milk* OR yield* OR perform* OR "production level*" OR "physiological status" OR "metabolic change*" OR "metabolic status" OR "mineral imbalance" OR "oxidative stress" OR lameness OR locomotion OR ketosis OR claw or claws OR hoof or hooves OR "somatic cell count*" OR mastit* OR dystocia* OR "retained placenta" OR "lung worm*" OR pneumonia OR "calving interval*" OR "insemination index" OR "Fat-to-protein ratio")	TS=("weak lamb*" OR hypothermia OR locomot* OR acidosis OR mastitis OR "somatic cell count*" OR "lung worm" OR "liver fluke" OR pneumonia OR scab* OR "lamb* interval" OR orf OR brucellosis OR footrot OR maedi OR clostridi* OR scrapie OR mycoplasma OR "fly strike" OR "pregnancy toxaemia" OR hypocalcemia OR hypomagnesemia OR dystrophy OR dermatophilosis OR enterotoxemia OR perfringens OR tetanus OR coccidiosis OR "poisonous plants")	TS=("post partum dysgalactia syndrome" OR "postpartum dysgalactia syndrome" OR mastit* OR farrow* OR oestrus OR estrus OR weaning* OR diarrhea OR diarrhoea OR lame* OR locomot* OR osteochond* OR arthritis OR erysipelas OR joint* OR leg OR legs OR "tail bit*" OR "skin lesion*" OR mycoplasma OR actinobacillus OR pneumonia OR enteritis OR pleurit* OR ascaris* OR "white spot*" OR growth OR root* OR forag* OR explorat* OR activity OR "active behavio*" OR "social interaction*" OR biting* OR aggressi*)	TS=("laying rate*" OR "mineral imbalance" OR leg OR legs OR lame* OR salpingitis OR "claw lesion*" OR parasit* OR nematode* OR coccidi* OR eimeria OR salmonella OR campylobacter OR mite* OR clostridia* OR mycoplasma OR ascites OR infecti* OR “injurious peck*” OR “feather peck*” OR cannibalism OR “keel bone*” OR fracture* OR aggressi* OR dustbath* OR “dust bath*” OR “sand bath*” OR sandbath* OR perch or perches OR scratch* OR thinning OR catch* OR virus* OR viral)

**Fig. 1:**
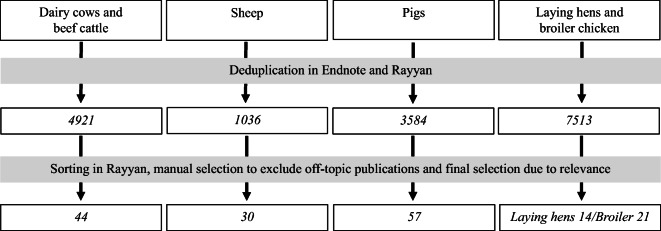
Flow diagram documenting studies included in the review

After a first search, the duplicated outcomes were removed, using the tool “deduplicate references” in Endnote, and again manually to double-check. Then, additional refining of the search results was performed using the screening tool “Rayyan” (Ouzzani et al. 2016). Publications that were obtained in the different searches were then sorted further in order to remove a large number of off-topic publications. The selection criteria for this sorting were set up prior to the search, and publications that were not about livestock or the specific animal categories were deleted. For this review, papers on non-relevant topics, i.e. nutrition, productive performance, product quality (e.g. weight gain, carcass traits, milk and egg quality) were also sorted out. Additionally, papers concerning genetic expression, breeding goals, life cycle analysis, environmental effects, public health, food safety, working environment, evaluation of biological indicators for different diseases, biosecurity and diagnostics were excluded. To enable a focus on studies conducted on production systems in similar economic contexts, studies performed or describing situations outside the European Union, North America or Canada were excluded. As a second step, all titles and abstracts of the search outcomes for each animal category, respectively, were read by the co-authors, and a further selection was made. Thus, relevant papers were identified and included in the review by co-authors with expertise in the different livestock species. The search and sorting process is presented in Fig. 1.

## Results—systematic search

### Database outcome

The literature search gave a large number of publications (17,056 in total), but throughout the review process, most of them were excluded as they did not fulfil the inclusion criteria (Fig. 1). After the final selection, in total 166 publications (44 publications on dairy and cattle, 30 on sheep and lamb, 57 on pigs, 14 on laying hens and 21 on broiler chickens) were relevant to include as results of the systematic search.

### Dairy cow and beef cattle production

The most common and important diseases investigated in organic dairy cows are mastitis, infertility, metabolic disorders and lameness. The number of studies addressing organic beef cattle is very limited and focuses on general health and welfare.

#### Mastitis

Udder health is the most commonly described health trait in scientific literature on organic dairy production since it constitutes one of the biggest health problems. Krieger et al. (2017) reported that the median prevalence for subclinical mastitis on 192 organic dairy farms in Germany, Spain, France and Sweden was 51.3% (interquartile range = 15.4). Different results have been reported for udder health when organic and conventional dairy herds were compared. An indirect indicator of udder health and mastitis is somatic cell count (SCC). When comparing SCC on organic dairy farms, with conventional farms, there are a lot of controversies at the different studies. There are findings of a positive deviation (Richert et al. 2013a; Levison et al. 2016), negative (Slagboom et al. 2016) or similar (Rodriguez-Bermudez et al. 2017) outcomes. Although SCC has been compared in organic and conventional systems worldwide, antibiotic usage has not been extensively taken into consideration. Studies that fail to consider other factors than the farming system (organic vs. conventional) could have caused or contributed to the reported differences. Factors explaining the higher cow milk SCC in organic farms could be avoidance of antibiotic treatments for mastitis, and lower milk yield in general, compared with conventionally kept dairy cows, in addition to differences in management practices (Schwendel et al. 2015). Besides, mastitis is the most frequently treated, recorded and mentioned disease in both conventional and organic dairy herds, and its therapy accounts for a very large proportion of the antibiotic drugs used in the farm. The lower treatment rate and, thus, reduced use of antibiotics may reduce antibacterial selection pressure. Garmo et al. (2010) investigated antibiotic resistance in udder pathogens from milk samples obtained from cases of clinical mastitis. They reported no difference between conventional and organic Norwegian red cows in quarter samples positive for mastitis bacteria and found that few *S. aureus* isolates resistance to penicillin in both management systems. Maintaining animal health without the use of therapeutic interventions is a major challenge for organic dairy farmers, including the reduction of the (preventive) use of antibiotics. For example, the approach to dry cow therapy (DCT) in organic farming differs from the practices on conventional farms. The necessity of application of DCT on cows in organic farms has however been addressed very little in research. Routine use of DCT is not allowed according to the organic regulations (and selective DCT is only permitted on individual animals, and cows will be treated after diagnosis), while it is a widespread habit in conventional farming systems in some parts of the world (Poizat et al. 2017). Bennedsgaard et al. (2010) concluded that antibiotic udder treatments may be reduced without apparent negative effects and that the control measures for SCC used on organic farms are at least as effective as those on conventional farms in controlling SCC. In this sense, preventive management practices are important in any dairy farm, but especially on organically managed farms. This is because the availability of products to treat a disease is limited (Stiglbauer et al. 2013), and all diagnostic measures and animal care at drying off aim to improve mammary gland health regardless of the farm system (Müller and Sauerwein 2010).

#### Metabolic/digestive disorders

The major interest regarding metabolic disorders in organic dairy production has been connected to the metabolic challenges in fulfilling the energy requirements of cows using forage-based diets. In a study of organic dairy herds in four European countries, the median prevalence for the risk of ketosis and acidosis were 10.0% (interquartile range = 7.7) and 3.2% (interquartile range = 4.7), respectively (Krieger et al. 2017). Concern has been expressed that many organic farms, particularly those that have converted recently, may have cows that are genetically selected for high milk yield and that an organic diet may not meet these animals’ requirements (Leiber et al. 2015). The major concern, however, is the risk of severe negative energy balance in cows in early lactation, due to the relatively low proportion of concentrate feed when following the organic legislation (Flaten and Lien 2009; Blanco-Penedo et al. 2012a). However, according to studies made by Blanco-Penedo et al. (2012a) and Richert et al. (2013a), there was no evidence that organic cows were metabolically more challenged or had a severe negative energy balance.

Animal nutrition in organic farming is highly dependent on local geographical conditions, and mineral deficiencies may occur in certain areas due to low mineral content or bioavailability of some trace elements in the soil. This can be associated with mineral imbalances in the diet due to the regulated restricted level of concentrate in the diet (EC 2008), although mineral feed additives can correct it. Despite this, organically managed animals could face an enhanced risk of mineral or nutritional deficiencies in areas with soil mineral deficiency. Compared to other farm systems, husbandry practices largely determine essential trace element status of organic livestock, as reported both for dairy (Blanco-Penedo et al. 2014) and beef cattle (calves and young cattle) (Blanco-Penedo et al. 2009). No signs of severe deficient concentration of essential elements have however been observed in organic dairy herds (Blanco-Penedo et al. 2014; Orjales et al. 2018) or organic beef farms (Blanco-Penedo et al. 2009). A high activity of the liver specific enzyme aspartate aminotransferase (GOT) in calves may indicate slight liver damage due to acidic conditions associated with the diet and may be a contributing factor of metabolic disorders in the fattening period. According to Blanco-Penedo et al. (2008), however, the GOT activity and activity of glutamate dehydrogenase and creatine kinase in beef calves from different production systems in Spain were within acceptable ranges, although a strong positive correlation was observed between the GOT activity and the proportion of concentrate in the diet.

#### Lameness and foot/hoof/leg disorders

Lameness is highly prevalent in today’s dairy and beef farming, and it negatively affects the well-being of animals. Numerous factors significantly influence the prevalence of lameness. According to Rutherford et al. (2009), organic management reduced herd lameness, due to the combination of different organic management practices. In their study, practices such as grazing and type of housing resulted in unique features determining the cows’ foot and leg health condition. On the other hand, some authors in the USA have stated that management factors differ significantly between organic and conventional dairy farms and that this is a confounding factor on the incidence of lameness. For example, a study on 292 herds showed prevalence of lameness ranging from 0 to 54% (mean 8%), and it did not differ among grazing systems (conventional non-grazing herds compared with organic and conventional herds with grazing practices) (Richert et al. 2013b). In another study by von Keyserlingk et al. (2017), the prevalence of lameness was lower on organic farms compared to non-organic farms, where fewer than 5% of the lactating cows had access to pasture during the grazing season. In European studies, prevalence estimates of lameness has been reported to range from 19 on organic farms in Germany (Leach et al. 2010) to 31% in Simmental dairy herds in Austria (Dippel et al. 2009) and 36% in UK herds (Barker et al. 2010). A recent EU project presented median herd prevalence of lameness in organic herds of 25% (range 0–51%), 20% (range 0–79%), 10% (range 0–27%) and 5% (range 0–25%) in France, Germany, Spain and Sweden, respectively (Sjöstrom et al. 2018).

Hock lesions in dairy cows are a common welfare problem. The prevalence of hock injuries on 40 organic and non-organic UK farms was on acceptable levels for cow comfort on many of the farms (Rutherford et al. 2008); however, organic farms had lower prevalence of hock lesions compared with non-organic farms (37.2 vs. 49.1%). Moreover, cows housed in free stalls with cubicles had a higher prevalence of hock lesions than those housed on straw bedding (46.0 vs. 25.0%). In a data set of 2922 lactating dairy cows (64 conventional and organic dairy farms with Holstein Friesian cows in Germany and 31 conventional dairy farms with the dual purpose breed Fleckvieh in Austria), it was found that the prevalence of integument alterations at hocks and carpal joints was high on all farms (Brenninkmeyer et al. 2016).

#### Respiratory diseases

Very little research has been performed on respiratory disorders in organic cattle and calves. No differences between conventional and organic dairy herds in prevalence of or incidence risk for certain viruses, such as bovine respiratory syncytial virus (BRSV) or bovine coronavirus (BoCV), have been described (Wolff et al. 2015). In organic beef cattle, results from logistic regression models of the appearance of the most common condemnations at Spanish slaughterhouses during 1 year revealed that organic calves had lower risk of lung condemnations compared with those from conventional farms (Blanco-Penedo et al. 2012b).

#### Parasite infections

Parasite infection constitutes one of the most important constraints on the welfare, health and productivity of grazing cattle in temperate regions (Charlier et al. 2009). Research in this topic has been performed across Europe in grazing systems with the major aims to assess prevalence and study the detrimental impact and the use of various diagnostic markers (Höglund et al. 2010; Ellis et al. 2011) and the impact of the infections on individual performance (May et al. 2017). The prevalence of liver fluke infection in a Swedish study performed 2008 was low, and it was only diagnosed in 7% of the105 organic and 6% of the 105 conventional herds (Höglund et al. 2010). The incidence, however, has increased dramatically since then (Novobilský et al. 2015). Organic farms did not have higher milk antibody levels for *Fasciola hepatica* than previous data reported from conventional farms in Spain (Orjales et al. 2017). Prevalence of helminth parasites in 114 organic herds in the USA were described to be low, based on low faecal egg counts (FEC) with only a few heifers with > 500 eggs per gramme faeces (EPG) (Sorge et al. 2015). In that study, egg counts of gastrointestinal parasites did not differ significantly between organic and conventional dairy herds, with the exception of significantly more strongyle-type eggs in organic compared to conventional herds (Sorge et al. 2015). According to a multivariable model approach in Sweden by Silverlås et al. (2009), similar prevalence of cryptosporidium was found in organic and conventional herds, both in dairy calves (44.7% vs. 52.3%) and cows (low due to development of immunity).

#### Behavioural effects

There is little research on the impact of organic regulations on cow welfare. No specific studies on the affective state or naturalness for organic cattle were found in this literature search. The relevance of behaviour in the context of naturalness and cow welfare is however brought up in the discussion of this review. Welfare and productive performance of dairy cows organically reared in plains or hilly areas in Italy was assessed with the animal need index ANI-35-L system (Bartussek et al. 2000) in a study by Martelli et al. (2010). The results indicated that, in the large majority of cases, organic dairy production successfully combines good levels of productivity, animal health and animal welfare. This was also found in Slovakian organic farms under extensive management (Kottferova et al. 2014). In a study on 30 low-input and organic dairy systems in three countries (using the Welfare Quality Protocol), the overall welfare state on the farms was acceptable (Kirchner et al. 2014). In general, that study showed that the weak points were related to the presence of injuries and discomfort of the lying areas of the cows. Specific problems such as mutilations, poor human-animal relationship or insufficient water provision were also identified on the studied farms.

Results from the USA on the welfare status on 192 organic dairy farms were, although with large variation, on similar level as the welfare status on 36 conventional dairy farms (Bergman et al. 2014). The results were below the desirable thresholds of many criteria of the assessment programmes currently used in the US dairy sector. In organic beef cattle, a small study based on the ANI-35-L/2000 system was conducted on four organic farms located in different regions of Lithuania. The farms had deep litter or cubicle housing, and the main outcome was an effect of housing, where deep littler was evaluated more favourably in comparison with cubicle housing (Stuoge et al. 2016). A study performed in the UK (Langford et al. 2011) compared the behaviour of cows in two different housing types (free stall with cubicles vs. straw-bedded pen) during and after peak feeding time. The results showed that the behaviour of organic dairy cows was not different from conventional dairy cows, and the results suggest that most behavioural welfare problems related to housing could be alleviated by management practices.

### Sheep production

The European Food Safety Authority (EFSA 2014) has reviewed health and welfare issues in sheep production. No discrimination between organic and conventional production was done, but across all systems, the most frequently identified welfare consequences for ewes were thermal stress, lameness and mastitis. Regarding lambs, thermal stress, pain due to management procedures, gastroenteric disorders and neonatal disorders were the main welfare consequences, and there were few differences among the systems of management. There is no evidence that disease problems are more severe or frequent on organic than on non-organic British sheep farms, according to Gray (2008). It is, however, not possible to make direct comparisons based on the general disease surveillance systems. No significant differences were observed between organic and conventional farms in terms of an animal needs index, as well as housing characteristics and animal-based parameters (e.g. dirtiness, overgrown hoofs, lameness, lesions, longevity), in an Italian study of 10 organic and 10 conventional sheep farms (Napolitano et al. 2009). The farms in both systems based their production on extensive grazing, and the authors hypothesize that the organic approach may be more important for the welfare of animals raised under intensive conditions, as compared to extensively reared sheep.

Kern et al. (2014) assessed animal health and body condition score (BCS) of 1562 ewes (6093 observations) on 20 German organic farms. On a remark point scale 1–5 (5 being without disorders), remarks 1–4 was found in 4.3% of the observations on legs/hooves, 4.5% on udder health and 2.6% on respiratory systems. Severe disorders (point 1 on the scale) were only found in 0.8% of the sheep regarding lameness, 3.0% for acute mastitis and 0.55% for obvious lung problems. The data was separated according to the primary purpose of the sheep, dairy, meat or landscape management, as the choice of breed mostly differs between the purposes, e.g. landraces being most common in extensive landscape management. A significant difference in BCS was found, with dairy sheep having a lower score than landrace sheep, probably because of poor nutrition.

#### Parasite infections

According to the studies mentioned in the section above, and several others (e.g. Cabaret and Nicourt 2009; Pilarczyk et al. 2008), parasitism by gastrointestinal nematodes (GIN) constitutes one of the biggest health problems in organic lamb production. Parasite eggs were found in 60% of 635 faecal samples from German organic sheep farms. More than one species was found in 15% of the samples. Strongyle nematodes and coccidians (*Eimeria* spp.) were the most common endoparasites, but also small lungworms (Metastrongylida) were found. The risk of being infected with GIN was highest for meat sheep compared to extensively held landrace ewes and sheep for dairy purpose, whereas the risk of being infected with *Eimeria* spp. was highest in dairy sheep systems (Kern et al. 2014). In comparisons of parasite burden between organic and conventional systems, there are varying results. In a recent Swedish investigation of 20 conventional and 19 organic farms, no significant differences in infection levels were observed between the systems (Höglund et al. 2019). *Trichostrongylus* spp. was the species with highest prevalence in both ewes and lambs, in both systems. The study also showed that *Haemonchus* is spread in large parts of Sweden.

In a Canadian study of 8 certified organic, 16 non-certified organic and 8 conventional farms, there was a general trend for sheep from the certified organic farms to have lower mean EPG, compared to the other farm types. In that study, the predominant nematode genera were *Teladorsagia*, *Haemonchus* and *Trichostrongylus*. There was a large variation in infection levels between individual sheep, where a few hosts had high FECs, while the majority had low or undetectable levels (Mederos et al. 2010). In a Polish comparison between production systems (three organic and two conventional farms), the results were the reverse (Pilarczyk et al. 2008). The mean prevalence of infection with internal parasites was 79% in sheep from the organic farms and 42% in those from the conventional farms. The prevalence of the protozoan *Eimeria* was almost double in the organic sheep. Also liver fluke and tapeworms were detected, on both conventional and organic farms. Liver fluke has been found in the UK to be an increasing problem that can be hard to control on organic farms (Gray 2008). In a Greek study of zoonotic parasites, no difference was seen for *Toxoplasma gondii* between organic and conventional farms (Kantzoura et al. 2013). In a similar study concerning risk factors for helminths and coccidia, no significant differences were observed between organic and conventional farms (Kantzoura et al. 2012). Control methods against GIN did not differ much between organic and conventional farms in a Swedish study (Höglund et al. 2019), and the majority of all the studied farms had used anthelmintics in the latest year. In addition, ectoparasites can be a problem. For example, according to Gray (2008), sheep scab in the UK is harder to handle in organic than in conventional production, due to the restrictions in use of medication.

#### Mastitis

One of the most important diseases in sheep husbandry is mastitis, and the SCC of milk can help identifying udder infections. In a German study, milk samples from 614 organic ewes with different primary purpose (dairy, meat or landscape management) were used to detect factors influencing SCC and assess risk factors that enhance the occurrence of bacteria in milk (Kern et al. 2013). The most common bacteria found were Staphylococcaceae (55%) and Streptococcaceae (23%). A log transformed SCC score was significantly lower in the extensively kept landrace ewes compared to ewes in meat and dairy systems. However, only 5% of all ewes had clinical mastitis. It was also found that meat sheep had the highest risks of udder problems, measured as occurrence of bacteria. Compared to dairy sheep, meat sheep raise their lambs, which could mean a higher stress for the udder, compared to milking. Ewes with two lambs had higher SCC than animals with only a lamb, probably due to higher sucking frequency. In addition, multiples sometimes suck from several dams, which increase the risk of spreading of bacteria between udders. In the review by EFSA (2014), mastitis was identified as an important welfare consequence, but it was identified mainly in dairy sheep, and it was stated that the occurrence is also affected by genetic factors. A tendency to lower bacteria count and SCC was identified in milk from organic farms in a Greek study including 25 organic and 25 conventional sheep and goat farms (Malissiova et al. 2017). This difference was probably due to differences in the hygienic farming practices, although there were no differences regarding the occurrence of *Staphylococcus aureus* and *Escherichia coli*. The study concluded that the milk from the organic farms had a better microbiological profile compared to that from the conventional farms.

#### Lamb mortality

Lamb mortality is not only costly but also an ethical issue. For the farmers, the loss of a single lamb or twins may be considered as negative, but deaths among triple lambs probably are more easily accepted. In a French study by Cabaret et al. (2011), the lamb mortality was high in both organic and conventional meat sheep farms. Also according to Verkaik (2011), lamb mortality on Dutch organic dairy sheep farms was relatively high, as the farmers rely much on self-reliance of the newborn lambs to save working time. Benoit et al. (2009) found that three lambings in 2 years in organic production were a risk factor for reproduction performance and health and thus, less sustainable, compared to one lambing per year. The system led to higher lamb mortality due to more stillbirths (toxoplasmosis) and higher numbers of digestive-tract strongyles and coccidia.

#### Lameness

The risk ratio of lameness was lower on organic farms than non-organic, according to a survey among 1260 English sheep farmers (Winter et al. 2015) with around 5% organic farms included. Sheep can be lame of many different reasons, e.g. interdigital dermatitis, foot rot or contagious ovine digital dermatitis (CODD). In 2016, CODD was estimated to be present in approximately 58% of the English sheep flocks, and the prevalence of CODD on organic farms was estimated to be 0.71, if it was 1.0 on a non-organic farm (Dickins et al. 2016).

According to a German study on organic farms, meat and dairy sheep had lower risk to get hoof problems compared to landrace sheep. Landrace sheep are common in rough environments, which may pose a higher risk for injuries (Kern et al. 2014).

#### Behavioural effects

In organic production, the space per animal often is larger than in conventional systems, and several studies support the fact that space is important for sheep behaviour and welfare. Increased animal density results in a reduction of space for locomotion, and greater number of animal interactions, especially of aggressive ones (Centoducati et al. 2015). According to Hansen (2015), increased indoor space contributed to better animal welfare, as indicated by increased lying time, more synchronized lying behaviour, less displacements and higher milk yield in sheep. An increasing indoor space allowance from 0.75 to 1.50 m^2^/ewe had positive effects on activity and behaviour in pregnant ewes, but a further increase to 2.25 m^2^/ewe had limited effects (Vik et al. 2017). When comparing indoor allowances between 0.5 and 1.5 m^2^/ewe, it was found that the total time spent lying down was lower, and standing was higher when the area decreased (Centoducati et al. 2015). In organic production, sheep often are outdoors more than in conventional systems. When assessing the effects of providing ewes with free access to an outdoor area compared to rearing indoors with equal space allowance, it was found that access to an external paddock had beneficial effects on immune reactivity and behavioural activities of lactating ewes (Caroprese 2008).

### Pig production

#### Metabolic/digestive disorders

Thinness or poor body condition of organic sows is a main concern in several European countries (Simoneit et al. 2012; Dippel et al. 2014; Früh et al. 2014). Weissensteiner et al. (2018) found that sows with larger litters had lower feed intake and greater weight loss during 1–2 weeks postpartum, when fed diets with high proportion of home-grown ingredients with low protein content. No effect of diet was however seen among sows with smaller litters. On the contrary, Kongsted and Hermansen (2009) found that even with a lactation length of 7 weeks or more, it was possible to avoid poor body condition at weaning in organic sows in Denmark. In addition, sows of native breeds managed under organic outdoor and indoor conditions in Poland showed satisfactory body condition (Szulc 2011). Poor body condition in fattening pigs was reported as a main problem in Danish herds (Früh et al. 2014), and studies on 101 organic sow herds in six European countries showed that diarrhoea in suckling and weaned piglets was a predominant disease (Sundrum et al. 2010). It was concluded that post-weaning diarrhoea is a relevant health and welfare problem in organic weaning and fattening pigs in many countries (Papatsiros 2011; Leeb et al. 2014; Früh et al. 2014), although, e.g. the UK reported diarrhoea as less frequent (Früh et al. 2014). Organic all-year-round outdoor systems (as compared with indoor systems with outdoor runs or semi-outdoor systems) lowered the frequency of diarrhoea (Leeb et al. 2019), and the access to roughage, such as silage, had a positive effect on pigs’ gastric health (Holinger et al. 2018b). In comparison with pigs only receiving straw, pigs that ate silage had an overall lower prevalence of gastric ulcers (score 6; 0.7% compared with 6.1%), and among the pigs with pathological damages, more severe damages, including gastric ulcers, were found in those that only received straw. The bibliography also indicates that inclusion of roughage may have potential to promote the immune competence of sows and their piglets (Werner et al. 2014).

#### Reproductive disorders and piglet mortality

According to Dippel et al. (2014), vulva lesions are a prevalent problem among organic sows. Reproductive disorders including MMA (metritis, mastitis, agalactia) were mentioned to be major health problems among organic sows in eight European countries (Früh et al. 2014). In addition, MMA was previously reported to be a predominant problem of organic sows in six European countries (Sundrum et al. 2010), with annual replacement rates of 32.4 ± 14.3%, indicating a high level of replaced sows, probably due to reproductive disorders, although the figures were lower than those from conventional farms. Piglet mortality is a relevant health and welfare problem in organic production. Sundrum et al. (2010) reported that mortality rates of organic piglets averaged 19.7 ± 9.7% and 4.9 ± 5.5% for pre- and post-weaning, respectively. In a study from 2007/2008 comprising 1200 litters from seven Danish organic sow herds, the mean total pre-weaning mortality amounted to as much as 33%, although ranging from 25 to 40% between herds (Sørensen and Pedersen 2013). A main concern is that a majority of deaths occurs within the first 4 days after farrowing, which affects the total number of weaned piglets (Lindgren et al. 2013; Prunier et al. 2014; Leeb et al. 2019), but according to Wallenbeck et al. (2009a), organic piglets in Sweden died at a higher age compared with conventional ones. Total loss of suckling piglets was found to be around 20% among 74 farms in eight countries, and did not differ between organic systems (outdoor all-year-round, semi-outdoor systems and indoor housing with outdoor run) according to Leeb et al. (2019). The number of stillborn piglets was found to be higher among organic sows compared to conventional ones, according to Lindgren et al. (2013). Large litters together with increasing parity were identified as risk factors for stillbirth (Rangstrup-Christensen et al. 2017) as well as for piglet mortality and early crushing of piglets in Danish organic herds (Rangstrup-Christensen et al. 2018a, b).

#### Respiratory diseases

According to the description of organic pig production in Europe by Früh et al. (2014), organic fattening pigs had better lung scores than conventional pigs in Austria, while respiratory diseases were described as main health problems for organic weaning and fattening pigs in Denmark, France, Germany and Switzerland. According to a review by Simoneit et al. (2012), pigs in organic indoor systems with outdoor access had respiratory illness in similar levels as those in conventional systems. All-year-round outdoor systems, however, showed lower prevalence of respiratory problems compared to organic semi-outdoor systems and indoor housing systems with outdoor runs (Leeb et al. 2019). The risk for chronic pneumonia and pleuritic in organic/free-range vs. conventional pigs was found to be equal, according to records on a large Danish abattoir (Alban et al. 2015). Airway infection was also the most prevalent disease complex (within-herd prevalence of approximately 20%) in organic and conventional free range systems, and did not differ from conventional indoor systems (Kongsted and Sørensen 2017).

#### Lameness/leg/foot health

Lameness of sows was less prevalent in organic compared to conventional herds (Dippel et al. 2014; Knage-Rasmussen et al. 2014) and less prevalent in all-year-round and partly outdoor sow housing systems compared to indoor systems (Leeb et al. 2019). However, according to Früh et al. (2014), all European countries involved in the study (Denmark, Germany, Italy, Sweden, Switzerland and the UK), reported leg problems (i.e. unspecified, injuries or joint problems) in organic sows as a major health problem. In the same study, leg and joint problems were identified as major health issues also among organic fattening pigs in some countries (Sweden, Italy and Germany).

The prevalence of auxiliary bursae (due to mechanical stress of the extremities) and injuries of claws was studied in 948 conventionally and 58 organically raised pigs, in four abattoirs in Southern Germany. Significantly lower and less severe prevalence of bursas and less injuries of claws were found among organically reared fattening pigs compared to conventional ones (Gareis et al. 2016). However, chronic infectious arthritis was more prevalent in organic/free range vs. conventional fattening pigs (Alban et al. 2015). It was suggested that this could be due to higher risk for erysipelas infections (*Erysipelothrix rhusiopathiae*), poorer hygiene (thus a higher general infection pressure) or more mechanical stress and joint injuries, predisposing the joint to arthritis. Lindgren et al. (2014) found more joint lesion slaughter remarks in organic pigs. Kongsted and Sørensen (2017) also found that organic and conventional free-range systems, with a larger space allowance and outdoor access, increased the risk of arthritis prevalence compared to conventional indoor systems. Further, Etterlin et al. (2014) found higher prevalence and severity of osteochondrosis lesions in the elbow and hock joints of fattening pigs allowed to range freely, compared to pigs kept in confined housing. However, even though free ranged (e.g. comparable with organic housing) fattening pigs had higher prevalence and more severe lesions than indoor confined pigs, lameness was not detected at a higher level. This indicates that the pigs may be less clinically affected as exercise may help strengthen the joint supportive tissues and lower the pain (Etterlin et al. 2015). According to Wallenbeck et al. (2020), the total incidence of joint rejections at slaughter was very low among Swedish organic slaughter pigs (1.3%), but the proportion of pigs with non-normal locomotion and lameness was high at 24 w of age (33.7 and 25.2%, respectively) and was increasing from w 13–24. In combination with the use of modern pig breeds, extensive and outdoor housing systems might be an underlying risk for leg health problems. The results from Wallenbeck et al. (2020) did however not support any evidence that leg health in Swedish commercial organic herds would be improved by changing sire breed from the commonly used sire breed Hampshire to Duroc.

#### Parasite infections

Endoparasites are common and more prevalent among organic pigs or pigs with access to outdoor areas, compared to conventional indoor pigs (Früh et al. 2014; Lindgren et al. 2014; Alban et al. 2015; Katakam et al. 2016; Kongsted and Sørensen 2017). Especially, higher level of *Ascaris suum* infections is common both before and after weaning and consequently with a higher prevalence of milk-spotted liver in organic pigs at slaughter. In spite of a more professional management of organic herds during the 1990s up to 2000, Leeb et al. (2014) found that organic weaners in Denmark were still heavily infected by *A. suum*. In an Austrian study, 69.5% of faecal samples from organic fattening farms were positive for *A. suum* (Kreinocker et al. 2017). According to Roepstorff et al. (2011), the long-lived eggs of *A. suum* and *T. suis* are a great challenge in organic pig herds and that even a 2–3-year pasture rotation programme may not be enough. Larger scale production systems based on indoor housing could further favour helminth transmission (Roepstorff et al. 2011). In addition, ectoparasites have been reported to be a general health problem on organic farms both in Austria and the UK (Früh et al. 2014). Lindgren et al. (2014) confirmed that mange, *Sarcoptes scabiei*, was the most important ectoparasite, causing skin lesions, restlessness and itching. In contrast, Leeb et al. (2019) observed very few signs of ectoparasites in a study with different systems (all-year-round outdoor, partly outdoor and indoor with outdoor run) in Austria.

#### Lesions and abscesses (skin, tail, body) and contagious diseases

Scar/hock lesions, abscesses in leg/toe, hernia and pyemia were found to be of lower risk in organic/free range compared with conventional systems, in a comparison of lesions found during meat inspection in Denmark. Abscesses on the mid and hind part of the body, old fractures, osteomyelitis and tail lesions were instead found to be more frequent in organic vs. conventional herds, whereas abscesses in head and ears and fresh fractures were similar across systems (Alban et al. 2015). Similar results were found by Kongsted and Sørensen (2017), who reported higher incidences of several lesions, e.g. tail lesions, skin lesions, bone fractures, septicaemia and abscesses, but lower prevalence of leg swellings, hernia and hoof abscesses in organic and conventional free-range pigs, compared with conventional indoor pigs at slaughter.

Important emerging pathogens are *Clostridium difficile* and different salmonella infections that cause enteric and clinical diseases in pigs, followed by diarrhoea. A Dutch investigation, both on individual pig level and on herd level, did not find any difference between the prevalence of *C. difficile* in pigs derived from conventional or organic farming types (Keessen et al. 2011). Hoogenboom et al. (2008) reported incidence of salmonella in samples of organic pig faeces at similar levels as for conventional ones. Interestingly, at farms that had converted to organic production more than 6 years before the study, no salmonella was detected. This was supported by Gosling et al. (2018) who found lower prevalence of *S. typhimurium* in outdoor than indoor farms in Great Britain. According to Astorga et al. (2010), the prevalence rates for *Salmonella* spp. was in general low among Iberian pigs in free-range systems. Similarly, detection of antibodies for salmonella in faecal samples from 59 Austrian organic pig farms was low (Kreinocker et al. 2017). A significantly higher risk of infection of toxoplasmosis (*T. gondii*) was however found for pigs with access to pasture (Wallander et al. 2016). Most infections in swine are subclinical but can cause clinical signs in pigs of all ages with health disorders as a result.

#### Behavioural effects

In organic production, the use of farrowing crates is not allowed (EC 2008). The effects on sow and piglet behaviour in classic farrowing crates in comparison with alternative farrowing systems, such as loose housing, are well investigated and described in the literature, but no bibliography of differences between organic and non-organic systems was found, and therefore this is not covered by the search results. However, as it has a major effect on sow and piglet behaviour and welfare, the subject is included in the discussion of this review.

Several studies on the positive effects of additional roughage (i.e. not only straw) and increased space allowance are present in the literature. A high fibre diet have been shown to increase satiety in growing pigs (Kallabis and Kaufmann 2012), and pigs with access to additional roughage such as grass, clover, chicory, maize or whole crop silage had greater opportunity to perform species-specific behaviours. Research has proven higher activity levels, and more time spent on foraging and rooting behaviours among pigs that received these substrates, compared to those that only received straw (Høøk Presto et al. 2009; Jensen et al. 2010; Holinger et al. 2018a; Presto et al. 2013; Presto Åkerfeldt et al. 2019). Roughage also occupied the pigs for longer time, resulting in fewer wounds on their bodies from violent social interactions (Presto et al. 2013), less aggressive interactions with other pigs in the lying area and less behaviours directed towards pen fittings (Høøk Presto et al. 2009; Jensen et al. 2010; Presto Åkerfeldt et al. 2019). A higher space allowance modified the pigs’ behaviour positively (Cornale et al. 2015) and influenced pigs to manipulate with the offered rooting material more often (Jensen et al. 2010). Additionally, the lower stocking density reduced the corticosteroid levels in pig faeces, which could indicate an improvement in welfare conditions (Cornale et al. 2015). Increased space allowance by outdoor rearing of heavyweight pigs increased their activity with a wider range of behaviours and was also found to lower the aggressions during preslaughter mixing, which further suggests improved welfare (Terlouw et al. 2009). Arroyo et al. (2019) showed that the neurophysiology of pigs was noticeably changed due to housing conditions (indoors vs. access to pasture) and road transport (high-stress vs. low-stress conditions), and it was suggested that animals raised partially outdoors respond differently (in a positive manner) to transport-related stress and can cope with new environments better than animals raised indoors. Outdoor areas with pasture increased the pigs’ activity level and their time spent in the outdoor range. Higher activity, more foraging and rooting behaviours, as well as fewer social interactions and tail manipulations were found among outdoor-housed pigs compared to conventional indoor-housed pigs (Høøk Presto et al. 2008). Correspondingly, pigs with access to a pasture area spent 21% of their time there and less time inside the pig house and on the concrete outdoor area, compared with pigs without pasture (Botermans et al. 2015).

In organic production, it is common to keep loose housed sows in groups on larger areas during lactation. This allows the sows to leave the piglets for shorter periods. When piglets were separated from the sow for 8 h/day 1 week prior to weaning, piglet feed consumption and growth in the immediate post-weaning period were improved. In combination with comingling with another litter, it also increased the creep feed intake and reduced the aggression level after weaning (Turpin et al. 2017). Mixing of piglets during lactation was beneficial for piglets’ social development, their adaptation to post-weaning situations and post-weaning performance (van Nieuwamerongen et al. 2014, 2015; Verdon et al. 2016, 2019). In a study by Bohnenkamp et al. (2013), early mixing had no effect on piglet growth but reduced agonistic behaviour and lesion scores in the piglets 2 days after weaning. Piglets that were socialized during lactation were also more “sleepy/tired” or “content/relaxed” than un-socialized pigs which were more “active/curious” or “aggressive/dominant”. This suggests that the socializing that often occurs in organic systems may be beneficial from a welfare perspective of piglets (Morgan et al. 2014), but the time for mixing is essential in order not to adventure piglet health (Thomsson et al. 2016).

### Laying hens and broiler chicken production

The main health challenges in organic laying hens have been found to be similar to those of loose housed laying hens indoor, as well as to free-range hens outdoor that are not managed according to the organic regulations (Hartcher and Jones 2017). The main areas of concern are injurious pecking (incl. feather pecking and cannibalism), internal and external parasites and possibilities to express species-specific behaviours. For organic broilers, the main health issues have been found to be foot pad dermatitis (FPD) and hock and breast lesions (van de Weerd et al. 2009). Van de Weerd et al. (2009) concluded that welfare problems are associated with suitability of breed, in particular in broilers, with nutritional challenges in relation to the banning of synthetic amino acids and also with range use and group size. They also found that there is a considerable variation in farming systems within the organic sector regarding farm size, housing and quality of the free-range area, capacity to produce home grown feed, opportunities for pasture rotation, etc., which influence the health and welfare conditions on individual farms.

Hygiene measures in houses and rotation of outdoor areas are important for all livestock species, specifically organic layers and broiler chickens that are exposed to wild birds that can transmit bird-specific or zoonotic diseases, e.g. avian influenza or salmonella. However, salmonella may not be as easily transmitted to free-ranged birds as previous thought, due to preventive measures (e.g. continuous salmonella monitoring, heat treatment of feed and no feeding of birds outdoors), which are effective strategies, lowering the prevalence (Wierup et al. 2017). Furthermore, the risk of nematode infection is decreasing if the birds have more access to the range (Thapa et al. 2015).

#### Metabolic/digestive disorders and productivity

Increased mortality due to injury and disease is often found among organic hens (and other free-range hens) compared with laying hens housed in aviaries or in cages (Leenstra et al. 2014). In organic broiler systems, production is significantly decreased for similar reasons, and long rearing times have also been found to relate to high mortality in broiler chickens (Rezaei et al. 2018). For poultry, the ban on synthetic amino acids in organic systems reduces the yield potential of the conventional hybrids (Eriksson et al. 2009; Eriksson et al. 2010). Improved and well-balanced diets to raise yields can also improve animal welfare by, e.g. preventing injurious behaviour and avoiding nutrient deficiencies. For example, problems with feather or vent pecking in laying hens were reduced by feeding an optimal diet with high-quality protein and roughage allowances (Rodenburg et al. 2013).

The correspondence between farm production system and breed/genetic potential has been extensively covered by previous research. Although traditional breeds are used in organic flocks in some countries, e.g. Italy, the same fast-growing hybrids as in conventional broiler production, i.e. Ross or Cobb from crosses of Cornish and White Rock bird strains, have been used in, e.g. the Nordic countries. These fast-growing and highly efficient broilers reach market weight in 5–6 weeks (ROSS performance objective, 2019). Rearing these birds for the longer period that organic rules demand (i.e. ≥ 70 days) increase mortality and culling rate due to severe leg weakness associated with their rapid growth (Eriksson et al. 2009). However, several factors have to be considered in the comparison of bird welfare when comparing organic and non-organic systems, such as housing conditions and genetics. Castellini et al. (2016) found that Ross chickens did not appear to be adapted to the organic system. They found that chickens with the highest daily weight gain had a negative linear correlation to adaptation to the system, whereas slow-growing strains with intermediate growth results showed the best adaptability index, and this is similar to the findings in previous studies (Castellini et al. 2012; Castellini et al. 2016). In a study that synthesized data from the Netherlands, the UK and Italy, several aspects of animal welfare, as well as performance, were assessed. It was found that an intermediate system had higher welfare score than extensive outdoor or organic systems, although the conventional systems had lowest animal welfare score (Gocsik et al. 2016). Furthermore, in a Belgian study (Tuyttens et al. 2008), it was found that the welfare of slow-growing broiler chickens in organic farms was improved compared to the welfare of fast-growing hybrids in conventional farms. In particular the lameness and other leg problems were more frequent in broilers from conventional farms (Tuyttens et al. 2008).

Concerning feather conditions, slower growing hybrids showed the best values for all considered body regions, as well as the absolute absence of foot pad and breast blister lesions (Castellini et al. 2016). This was also confirmed by Skomorucha and Sosnowka-Czajka (2017), who found that Ross 308 chickens are probably the least suitable for rearing during summer production cycles, as these birds had low ability to adapt to warmer conditions.

#### Lameness, foot pad dermatitis and other diseases

Positive effects from free-range systems on foot and leg health are expected in layers, as well as in broiler chickens. However, there are no clear correlations demonstrated yet, as choice of bird strain as well as different slaughter ages of broilers in conventional and organic production is influencing the results. FPD is more common in conventional production than in organic broiler farms where housing conditions are different and the stocking density is lower. In a study by Gouveia et al. (2009), broiler welfare of birds in extensive indoor systems (EI) and traditional free-range systems (TFR) during rearing and preslaughter handling was assessed by measuring post mortem lesions. TFR birds exhibited the highest prevalence of bruises and lowest prevalence of FPD. Furthermore, the study showed that lesions were associated with other factors than production system, such as distance to abattoir and gender of the birds. In a Danish surveillance study of FPD at slaughter, as an indicator of on-farm broiler welfare, Lund et al. (2017) found less FPD among organic than conventional broilers. However, the results were inconsistent, and the authors considered that organic broiler were more difficult to score than conventional broilers, which might explain the inconsistency (Lund et al. 2017). Bergmann et al. (2016) found that shortly before slaughter, 2.5% of the organic broilers (day 40) and 16.8% of the conventional broilers (day 35) showed various degrees of FPD, although factors like farm and bird strain also had a significant effect on the occurrence of hyperkeratosis and FPD. Furthermore, the live body weight had a significant effect on the prevalence of hock burn in both strains. The authors found that obvious lameness (0.8%) and immobility (0.5%) was only identified in conventional broilers, and not in any birds in organic production. Outdoor access and low-nutrient diet also resulted in better gait score according to Fanatico et al. (2008).

According to Tahamtani et al. (2018), lameness was less prevalent and severe in Danish organic broiler systems relative to conventional production. In a recent Swedish study by Wilhelmsson et al. (2019), the same trend was found for lameness as well as for other clinical health problems including mortality rate, contact dermatitis and plumage cleanliness. Indications of poor welfare were observed in the slower-growing hybrid compared to a fast-growing hybrid but to a lesser extent and later during rearing (Wilhelmsson et al. 2019). Sarica et al. (2014) found that FPD scores varied significantly between genotypes, with higher scores found in fast-growing chickens. Heavier birds and male birds were also found to have more problems, and chickens with outdoor access had higher FPD scores than those without outside access. In a study by Fanatico et al. (2008), it was found that genotype affected leg health, with slow-growing birds having better gait scores and less tibial dyschondroplasia. Free-range access may also have negative impacts by increased risk for diseases carried by wildlife, parasitic infections, predation and contact with soil contaminants (Newberry 2017). However, Wnuk-Gnich et al. (2016) found that broiler chickens having access to free ranging systems were characterized by a significantly lower mortality rate compared to the control birds. High levels of free-range use have been associated with a reduced incidence of keel bone fractures in laying hens (Richards et al. 2012; Jung et al. 2019). The literature search did not result in specific studies of the occurrence of erysipelas infections; thus, this was excluded in this review.

#### Parasite infections

Regarding internal parasites of organic poultry, helminths, such as gastrointestinal nematodes (GIN) and cestodes (tape worms), are mainly seen as a health and welfare problems in laying hens, whereas coccidiosis caused by *Eimeria* spp. is more prevalent in broiler chickens. In laying hens, the most important GIN are *Ascaridia galli* and *Heterakis* spp., and several studies have shown that free-range and loose-housed indoor flocks have higher numbers of *A. galli* eggs than caged flocks (Dao et al. 2019; Fossum et al. 2009; Jansson et al. 2010). In a British epidemiological study in farms with egg production in free-range areas outdoor, organic as well as non-organic, and with stationary as well as mobile houses, it was found that *A. galli* and *Heterakis* spp. were the most common intestinal parasites (Sherwin et al. 2013). However, they concluded that these infections were not severe as no negative effect on welfare indicators or production variables was found (Sherwin et al. 2013). Nevertheless, other studies have found that infection with *A. galli* and *Heterakis* spp. has been associated with increased mortality in organic egg production but may be reduced by control measures (Hinrichsen et al. 2016). In a trans-European study, Thapa et al. (2015) found that *A. galli* was highly prevalent across Europe (69.5% of all flocks). Furthermore, they found that the prevalence of cestodes of *Raillietina* spp. was 13.6%. The authors found, when analysing several management risk factors, that only pasture access time had a significant negative association with worm burden from *A. galli*. This was in contrast to the previous belief that outdoor access may increase the risk of helminth infections in production animals. Jansson et al. (2010) found no significant difference in prevalence of *A. galli* between hens kept on litter indoors and free-range/organic hens. Furthermore, they found that absence of a hygiene barrier at the entrance of the unit was a risk factor for increasing transmission of GIN. This suggests that parasite infection was introduced horizontally to the farms.

No publications of coccidiosis in organic broiler chickens comparing the situation to non-organic free-range broilers were found in this literature review according to the selection criteria. As paraphyletic treatment with coccidiostats is not allowed in organic production, the birds are exposed to infection. However, vaccination is commonly used, and the decreased stocking density in organic broiler production as well as the restricted group size may be favourable in reducing the contamination risk. The red mite (*Dermanyssus gallinae*) is the most important ectoparasite of laying hens in Europe, and it is more prevalent in non-caged layer flocks, than in caged flocks. As the parasite infestation is mainly depending on housing equipment and management of indoor areas, the infestation risk is not different in production system where the hens are either loose housed indoor (i.e. aviaries, barn systems or housed in organic or non-organic free-range systems with outdoor access).

#### Behavioural effects

In comparison with aviary systems with high stocking density, the increased space allowance and free-range access in organic egg production are meant to improve the welfare of laying hens by providing them possibilities to express their species-specific behaviour. This can result in a lower incidence of maladaptive behaviours like feather pecking and cannibalism, if the hens use the outdoor area, as reported in different studies (Bestman et al. 2017; Jung et al. 2019). The understanding of injurious pecking, however, still needs to be improved in order to fully prevent the problem. In a study by Yngvesson et al. (2017), 27% of the slow-growing broilers perched simultaneously at night, a very low proportion compared with, e.g. laying hens. The authors suggested that to ensure acceptable welfare and health in these broilers, it may be necessary to provide more opportunities for them to rest in an elevated position. Bozakova et al. (2011) found that higher welfare of birds reared organically was linked to the greater number of birds spending their time dust bathing and preening, fewer episodes of aggressive behaviours, as well as lower plasma corticosterone levels, compared to birds reared indoor on litter. In stress tests, it was found that slow-growing bird strains (more commonly used in organic production systems) displayed a quicker reaction time when submitted to tonic immobility test, which implies less stress reaction (Castellini et al. 2016). In the same study, they also observed a greater variety of the behaviour of slow-growing birds and that they exploited all the pasture area, compared with medium-slow and fast-growing birds.

## Discussion

### Impact on the systematic search on the outcome

Systematic reviews are important tools to summarize data correctly and reliably and should include a well-defined question that is quantitatively analysed. We aimed to describe the status of animal health and welfare in organic animal production. Therefore, the method of the current paper would be better described as a systematic mapping, based on a systematic search. Using the PIO approach, with the same search items in different search engines and in collaboration with a professional librarian specialized in scientific databases, gave a homogeneous and reliable search result for the different animal categories. However, the diverse and non-consistent use of the word “organic” in the literature made the search very extensive in order to capture relevant publications, which was reflected in the large number of publications.

The included health and welfare indicators for the different animal categories aimed to reflect relevant health issues and behavioural effects and were selected based on current knowledge and previous research. The study, which was limited to the most important species in organic production, i.e. dairy and beef cattle, sheep, pig, laying hen and broiler chicken, had the starting point for the search period in year 2008. This point was set in order for comparison between systems under similar conditions reflected in more equivalent production practices, as the new commission regulation was implemented at that time. In accordance with the reviews by Hovi et al. (2003) and van Wagenberg et al. (2017), the present review also found that the included studies varied broadly due to different research criteria, variation in the number of farms and differences in farm production and conditions as well as management systems. This might have had an impact on the outcome. It was however not our intention to assess the methodological quality of the reviewed studies, but due to the descriptive nature of some literature, low sample size in some studies or lack of available data, it was not possible to compare the studies in the same domain and evaluate the benchmark appropriately.

### Health status and behavioural effects in organic production

#### Dairy cow, beef cattle and sheep production

The outcome of this review indicates that the main health issues facing organic dairy farmers are basically similar to those reported on non-organic farms. However, it would be dangerous to rely on this body of evidence alone, given the almost certain differences in reporting health and treatments from organic and non-organic farms (Sutherland et al. 2013). There is no apparent simple relationship between single factors and the prevalence of mastitis in dairy cows and sheep in the organic context. However, genetic factors, production type, management and hygienic farming practices are reported as risk factors (EFSA 2014; Casao et al. 2017). Various studies performed in several European countries have shown that current udder health levels implicate necessary improvement in organic dairy farms (Ivemeyer et al. 2012; Krieger et al. 2017). Lamb mortality is an important welfare issue although there is little evidence of its relation to specific factors in an organic context. The literature indicates that organic management can reduce lameness in dairy cow and sheep herds. The prevalence of lameness is on the other hand significantly influenced by numerous factors, and the combination of management practices, including grazing requirements that are combined with different types of housing, will determine the foot and leg health conditions (Pinedo et al. 2017). Obvious lameness indicates that the animal has severe pain. Gait assessment could be used as an indirect measure of foot disease, thus representing a valid welfare parameter (Napolitano et al. 2009), and recording of the prevalence of hock lesions and footbath should be used in order to keep the disease load low. Parasitism by GIN is regarded to be one of the biggest health problems in organic lamb production although there seems to be a large variation in parasite load on an individual level as well as between farms. In order to lower the prevalence, different alternative measures to control GIN is recommended (Burke et al. 2012), combined with breeding for resistance (Hooper et al. 2014; Chevrotiere et al. 2011; Burke et al. 2012; Williams 2011). There is a well-documented genetic variation in resistance to GIN, and breeding for developing genetically resistant sheep is a promising strategy (Mederos et al. 2012).

The effects of roughage, larger areas and outdoor access on the behaviour pattern among organic dairy cows and sheep are according to the findings of this review, not that pronounced. This is probably due to smaller differences in the appliance of these systems, compared with those of monogastric animals, as both organic and conventional dairy cow and sheep production systems, grazing and forage-based feeding are common. Although some publication have found positive effects on behaviour, due to factors such as larger areas and longer outdoor periods, this review could not show clear evidence of a positive health and welfare effect, on, e.g. organic than on non-organic sheep farms (Gray 2008; Napolitano et al. 2009). The same situation seems to be the case in organic dairy farms, where the welfare status has been shown to be at a similar level as in conventional dairy farms (Bergman et al. 2014). However, in comparison with feedlot systems, where, e.g. lambs are fattened with large amounts of concentrates in crowded pens and barren environments, health and welfare problems however seem less probable to develop in organic production. In spite of differences in pasture use, feeds and feed additives, breed and age distribution and reproductive management across farms and production systems, it is suggested that lack of behavioural welfare benefits relate to housing and thereby could be improved by management practices (Langford et al. 2011).

#### Pig, laying hen and broiler production

Piglet mortality due to the crushing of piglets and inadequate nursing of group-housed piglets are still the most frequently reported causes of death across organic production systems (Lindgren et al. 2013; Westin et al. 2015). Large litters, especially in sows with higher parity number, and farrowing system (loose housing and group housing with free farrowing) are the major risks for stillbirth (Rangstrup-Christensen et al. 2017, 2018a, b) and crushing of piglets (Hales et al. 2014, 2015; Grimberg-Henrici et al. 2019). The primary reason to use farrowing crates is piglet survival. The crates do however prevent sows from moving freely and interacting unrestrictedly with their piglets and are therefore banned in organic production. In the context of animal health, and the opportunity for animals to live a natural life according to their physiological and behavioural conditions and well-being, the organic sector is facing a clear goal conflict within this area. Osteochondrosis may represent a larger health and animal welfare problem in organic outdoor and free-range pig production than previously assumed. Although exercise might help to strengthen the joint supportive tissues, the literature review indicates that pig leg health is challenged during rearing in environments with large space allowances and outdoor access and point at the importance of preventing and monitoring leg health in such production systems (Etterlin et al. 2014, 2015; Wallenbeck et al. 2020). Parasite infections and respiratory diseases do not seem to differ considerably either between organic and conventional production or different housing systems within organic production according to the literature. As organic farming in recent years is facing new indoor-based housing environments, this might be one causative factor to the results.

Organic egg production is predominantly challenged by health and welfare problems related to injurious feather pecking and cannibalism, as well as internal and external parasites, and the possibilities for birds to express their species-specific behaviours. There are studies highlighting higher mortality related to the production system (e.g. organic and free range), but the reviewed data demonstrates that the health and welfare challenges are the same as those in non-organic systems, independently of indoor or (non-organic) outdoor free-range systems (Hartcher and Jones 2017). For organic broilers, identified and relevant health parameters that need improvement are dermatitis of footpads, hocks and breasts. The welfare problems among organic broiler chickens have a clear connection to the suitability of breed, with nutritional challenges, metabolic disorders and leg problems related to the banning of synthetic amino acids, use of outdoor areas and group size (van de Weerd et al. 2009). The high incidences of mortality due to lameness and circulatory problems reported for birds of fast-growing breeds reared in organic production systems with long rearing periods further reduce the net yield (Wallenbeck et al. 2017; Rezaei et al. 2018). If future organic regulations were to allow supplementation of essential amino acids in livestock diets, that would be a major advantage, allowing avoidance of nutrient leakage caused by overfeeding (Eriksson et al. 2010; Leenstra et al. 2014). As an alternative to fast-growing hybrids, slower-growing genotypes could be more suitable for production systems with longer rearing periods. Additional scientific information about the effect of low-protein diets on growth patterns for slow-, medium- and fast-growing genotypes to that of Fanatico et al. (2005) are needed.

Access to additional roughage, high fibre diets, increased space allowance and outdoor and free-range access enables better opportunities for animals to perform species-specific behaviours and live a natural life and are therefore included as legislative rules. The literature reviewed clearly demonstrates that these elements have positive effects on the behaviour of organic pigs and poultry. The findings are not surprising, as these organic production systems differ substantially from the praxis in non-organic pig, layer and broiler production systems. Moreover, keeping sows in loose housed farrowing systems enhances sow behaviour and allows for improvements in post-weaning piglet feed consumption and growth. Among organic layers, the incidence of harmful behaviours, such as feather pecking and cannibalism, can be reduced when hens are reared in lower stocking densities and are offered free-range areas (Bestman et al. 2017; Jung et al. 2019). The use of slower-growing breeds in broiler production is a prerequisite for the longer rearing time and adaptation to the production system. It is a major impact for improved animal health at the flock level, due to more appropriate behaviour and is important, as it also will increase the number of broilers being healthy at slaughter compared with fast-growing breeds (Rezaei et al. 2018; Wallenbeck et al. 2017).

### Impact of the outcome in the context of the organic values

The current mapping indicates that varying results and inconsistent interpretations within the area of health and welfare in organic production still occur, which is in accordance with previous reviews (Hovi et al. 2003; Sundrum et al. 2010; van Wagenberg et al. 2017). Several publications reviewed in the present study address factors related to housing and management that negatively influence animal health but, on the contrary, have a positive effect on animal behaviour. A multifunctional farming system according to the fundamental organic principles might cause the system as such to imply conflicts interacting with animal health and welfare of the individual animal, e.g. leg health or parasite burden of free-ranging animals. Although sick animals should be treated with appropriate therapy, organic farmers are encouraged to use preventive measures to lower, e.g. the pathogen load, in order to eliminate future morbidity (IFOAM 2005). This conflict may be a causative factor to the criticism about animal welfare in organic production. Along with the development of organic animal husbandry, the risks for goal conflicts and practical implications, not foreseen when the legislative rules were developed, arises when farmers put the rules into practice.

Organic production aims for less intensive animal production, which generally means that the animals have access to a more spacious and enriched environment, access to an outdoor range as well as restricted group sizes (EC 2008). On the contrary, the organic sector has experienced an increased intensification in recent years due to economic reasons for the individual farm. Moreover, defined as a production method by minimum standards, doubts have arisen whether organic animal husbandry can be characterized in dissociation to or even achieve better than conventional animal husbandry systems do regarding animal health and welfare (Sundrum et al. 2010; Sutherland et al. 2013). Despite legally and well-defined basic rules of organic production, a broad diversity of production systems occurs, including large-scale production systems with a higher number of animals per farm. The rising demand for productivity and profitability among farmers, with, e.g. continued breeding for high growth rates, without taking other important breeding traits such as animal health and behaviour into account, and the use of modern high producing breeds, often selected for conventional production environments, in organic production might risk aggravating current health problems further (Röös et al. 2018).

Due to legislative rules, organic farmers (except in poultry) are more likely to keep the number of purchased animals low and, if they buy animals, these are kept in quarantine (Toma et al. 2013). This could be a potential risk for animals being kept longer in production, with impaired health as a result. Animals’ ability to live a natural life and perform species-specific behaviours is regarded as a prerequisite for good animal welfare; however, the combination of organic management practices with different types of housing results in characteristics that will affect animals’ health condition (Pinedo et al. 2017). Moreover, genotype × environment interactions indicate that modern high-producing breeds of pigs might not always be the best suited in an organic environment, e.g. outdoor areas, although it seems that the problems are less for high-producing laying hens and dairy cows (Wallenbeck et al. 2009b; Sundberg et al. 2010). Although health-related traits are included in selection indexes for livestock, inclusion of functional robustness traits will be important (Oltenacu and Broom 2010). For further improvement, the inclusion of, e.g. behavioural traits (Turner 2011) and non-market values, such as ethical values or environmental impact (Olesen et al. 2000) should be considered. Despite the counteracting relationship between robustness and production traits, a positive genetic trend in both could be achieved when they are appropriately included in breeding goals and selection criteria (Knap 2009). For this, improved identification of farm-specific factors and outcome-oriented animal health and welfare indicators is a prerequisite, and policy options with potential for securing a low prevalence of production diseases on organic farms need to be highlighted.

The outcome of the current mapping supports the fact that the health status of organic dairy cow, beef cattle, sheep, pig, laying hen and broiler chickens in general is good, in relation to the definition and guiding principles of animal health and welfare stipulated by the World Organisation for Animal Health (OIE 2019). In accordance with Sundrum (2001), the findings indicate that the minimum standards of organic farming seem to provide a basis for good living conditions of farm animals but appear to be insufficient to ensure a higher animal health status than in conventional livestock farming. In the context of the fundamental ideas of organic farming and animal welfare, where natural living and animals’ emotional states play important parts (IFOAM 2005), the positive behavioural effects found in this review indicate that the organic standards offer a good framework for high animal welfare management.

Although organic production meets the needs of positive welfare and naturalness, the systems are still facing several challenges, in particular related to animal health. The outcomes from this review support the conclusions by Hovi et al. (2003) that there is a need of progression regarding apparent goal conflicts that arise between the fundamental principles of organic farming and animal health and welfare due to management and practical implications. In an era that is turning from minimizing animal suffering towards focusing on positive animal welfare, it is recognized that the current reference standards in animal welfare science, an emerging field of research, requires the presence of positive experiences, as well as the absence of negative.

## Conclusions

The welfare status of organic dairy cow, beef cattle, sheep, pig, laying hen and broiler chickens, in general, is good, in relation to the definition and guiding principles of animal health and welfare. Although there are some areas with health and welfare advantages in organic livestock production systems, some still require improvements, and there is no strong evidence supporting that animal health and welfare are neither inferior nor distinctly better, in organic compared with conventional production systems according to the literature we explored. Mastitis in organic dairy and sheep farms, high mortality rates of lambs, piglets and broiler chickens, high parasite load and infestation, as well as hoof and leg problems among all animal species are relevant health issues that need improvement. Prevalence of diseases is mainly related to management practices, grazing and housing systems; therefore, possibilities to monitor health are important factors determining animal health conditions.

The possibilities for animals to perform species-specific behaviours seem to be better met by the organic regulation, which indicates that the organic standards offer a good framework for good animal welfare management, even though they appear to be insufficient to ensure a higher animal health status than in conventional livestock farming. In order to consider and guarantee health-related aspects of animal welfare, outcome-based assessments should be implemented in organic standards.
